# Control Work

**Published:** 1895-11

**Authors:** 


### CONTROL WORK.

*Australian Colonies and New Zealand.*—A proclamation by the Governor of Queensland and its dependencies to the Inspector of Live Stock for New Zealand and Australian Colonies at the port of San Francisco, under the Diseased Animal Act, (which provides for the prohibition or restriction of the importation of cattle, horses, sheep, goats, pigs, poultry, and other animals from such places as may appear necessary, looking to the better control of tuberculosis, glanders, and farcy), gives notice that all cattle landing on their territory shall be subject to a tuberculin-test, and all horses to a mallein-test, before final acceptance, and those found diseased will either be destroyed, returned, or so disposed of as may be deemed best. This proclamation was issued June 19, 1895, and the tests are to be made by duly-qualified veterinary surgeons.

*Italy.*—A letter from one of the correspondents of the *British Medical Journal* refers to the excellent regulations in force in Rome, relating to the health of the animals furnishing the milk-supply of the "Eternal City." We seem sometimes to have to go abroad for progress. "All milch-cows and other animals which supply milk in the suburbs and Agro-Romano will be subjected to a vigorous examination by the municipal veterinary surgeons. Notice of all animals introduced into the commune must be given to the health authorities before the milk can be sold, and it is then placed under the inspection of the veterinary surgeon. Healthy animals will be marked on the horn and a special license given to the owner. These animals are inspected every year, in the months of April, May, and June, and on other occasions when deemed necessary. Suspected cases of tuberculosis will be tested with tuberculin at the expense of the owners, and diseased ones slaughtered. These regulations will apply to the whole commune, thus assuring to all the inhabitants a security to which every city in the world is justly entitled."

Under the Act of March, 1891, amended March 2, 1895, Secretary Morton has issued an order to go into effect January 1, 1896, relative to meat inspection, commanding that all beef-products for exportation shall be accompanied by the certificate of an Inspector of the Bureau of Animal Industry, showing that the cattle were free from all disease and the meat sound and wholesome. This applies to all canned and barrelled meats, which must be labelled the species of animals contained. Meat not so marked will be opened for inspection. No vessels will be allowed clearance-papers with any package of meat unmarked. This is aimed specially at the diseased-meat exportation and will demand an increase of the number of inspectors, all of which will be grateful news to the ranks of the profession.

*Massachusetts.*—The *Boston Medical and Surgical Journal*, in speaking of the actions of the United States Veterinary Medical Association at Des Moines in indorsing tuberculin as the only diagnostic agent in detecting suspected cases of tuberculosis, urges upon the Legislature of that State the retracing of the backward step taken by them last spring.

*Iowa* (one of the newer fields to take up the consideration of tuberculosis) finds that she has in certain localities more of this disease to deal with than she had suspected. The tuberculin and milk-tests (examination microscopically for bacilli in milk) side by side varied extremely; the former proved reliable, the latter entirely unreliable, so far as determining the affected cattle of a herd.

*Louisiana.*—Charbon is said to prevail to a serious extent in parts of Louisiana among the mules. Vaccination is about to be undertaken there with the Pasteur vaccine preparations.

